# Effect of an Educational Intervention on Lower Limb Pain During the Hajj Mass Gathering: A Randomized Controlled Trial

**DOI:** 10.7759/cureus.117947

**Published:** 2026-10-14

**Authors:** Yasser Bouchouata, Latifa Tahiri, Salma Elboughabi, Safa Rakhami, Ayda Belmlih, Hajar Elgmiri, Saad Sihida, Nouhaila El-Maimouni, Meryem Kardoudi, Moncef Ziani, Hammou Jaouad, Amina Chrif Alaoui, Mustapha Oufqir, Khadija Fajjary, Fatima Jassab, Redouane Abouqal, Jihane Belayachi, Fatine Kronbi, Hanan Rkain, Mohammed Youbi, Samia Karkouri, Fadoua Allali

**Affiliations:** 1 Department of Rheumatology B, El Ayachi Hospital, Ibn Sina University Hospital, Rabat, MAR; 2 Department of Physical Medicine and Rehabilitation, El Ayachi Hospital, Ibn Sina University Hospital, Rabat, MAR; 3 Directorate of Epidemiology and Disease Control, Ministry of Health and Social Protection, Rabat, MAR; 4 Medical Delegation, Ministry of Health and Social Protection, Sale, MAR; 5 Laboratory of Biostatistics, Clinical Research and Epidemiology, Public Health Department, Faculty of Medicine and Pharmacy of Rabat, Mohammed V University, Rabat, MAR

**Keywords:** lower extremity, mass gatherings, musculoskeletal pain, patient education, primary prevention, randomized controlled trial

## Abstract

Introduction/objectives: Lower limb pain is common and disabling from age 50 onwards, yet prevention before predictable episodes of intense ambulatory load has never been tested. We evaluated a pre-departure therapeutic patient education program in pilgrims attending a mass gathering.

Method: A parallel-group randomized controlled trial (1:1) was conducted in two Moroccan vaccination centers: 218 pilgrims aged 50-75 years were enrolled in April 2025 and followed until late June 2025. The intervention group received a therapeutic patient education program, comprising a one-hour group education session plus an unsupervised home-based adapted physical activity program and WhatsApp reminders; controls received none. The primary outcome was lower limb pain on a visual analog scale (0-10) 48 hours after the end of the peak activity period. Analysis was by intention to treat, using linear mixed models, with blinded assessors and analyst.

Results: The primary outcome was available for 216 of 218 participants (mean age: 62.6 years; 147 (67.4%) women). At T2 (48 hours after the end of the peak activity period), lower limb pain was lower in the education group (−1.75 points; 95% CI: −2.40 to −1.09; p<0.001); a smaller difference was already present seven days before departure, and the difference persisted seven days after return. The education group also had less limping (24 (22%) versus 42 (39.3%); p=0.01), less increased analgesic use (33 (30.3%) versus 48 (44.9%); p=0.027), less foot pain (11 (10.1%) versus 24 (22.4%); p=0.01), and better quality of life (+0.059; p=0.021). Low back pain, healthcare use, and falls did not differ.

Conclusions: A pre-departure therapeutic patient education program, evaluated as a whole, produced a statistically significant reduction in lower limb pain in adults aged 50-75 years exposed to several days of intense walking. The point estimate did not reach the 2-point threshold set a priori, so a clinically important benefit can be neither confirmed nor excluded. Predictable episodes of high ambulatory load may offer a practical window for resource-light prevention, which requires confirmation against an active comparator.

## Introduction

Musculoskeletal conditions are the leading cause of disability worldwide, affecting around 1.71 billion people, and their burden rises steeply with age [1]. Lower limb pain, whatever its cause, is frequent from midlife onwards, reported by more than 40% of adults aged 45-64 years and about half of those aged 65 years and over [2]. Such pain threatens walking capacity and participation, so measures able to prevent it before it occurs have value beyond the treatment of established disease.

Adults of this age nevertheless undertake, at predictable and dated moments, episodes of intense and unaccustomed ambulatory load, and organized mass-participation travel is a prototypical example. The annual pilgrimage to Saudi Arabia, one of the largest recurrent mass gatherings in the world, brings together several million participants each year [3,4], with Morocco contributing an annual contingent of around 34,000 [5] in whom metabolic comorbidity is frequent [6]. The itinerary imposes an intense physical load: participants walk 5-15 km per day and up to 63 km over the stay [3], often well above their habitual activity level [7]. Musculoskeletal pain is consequently frequent and preferentially located in the lower body, with an overall prevalence of 80.5%, of which 65.4% involved the lower limb [3], and a similar figure of 78.6% in 2022 [8].

Current management remains reactive and mainly pharmacological. Analgesics and nonsteroidal anti-inflammatory drugs (NSAIDs) are among the classes most often prescribed in outpatient care in this setting [9], and much of this consumption escapes healthcare structures: many participants do not consult for pain they judge moderate [3] or use drugs brought from their own country [9]. Amid heat, prolonged effort, and dehydration, the risk of misuse is real, particularly for NSAIDs [10,11]. Existing preventive recommendations, by contrast, remain descriptive and unevaluated [3,8].

Therapeutic patient education is a plausible preventive alternative, with established efficacy in chronic diseases [12]. Its effect on musculoskeletal pain remains debated: it is uncertain when education is delivered alone and becomes clearer when combined with another treatment, particularly exercise [13]. No randomized trial has evaluated a structured education program delivered before an anticipated episode of high ambulatory load, so its effect on lower limb pain, low back pain, and analgesic self-medication remains unknown. This gap matters because the pre-departure consultation is a systematic, already organized contact with a large population, never yet used for evaluated educational purposes.

We carried out a randomized controlled trial to determine whether a structured pre-departure education program reduced lower limb pain, rated on a visual analog scale (VAS) (0-10) 48 hours after the end of the peak activity period, in pilgrims aged 50-75 years. Secondary objectives concerned low back pain, the location of maximal lower limb pain, quality of life, increased analgesic use, healthcare use, limping, muscle cramps, and falls.

## Materials and methods

Study design and setting

We carried out a two-arm parallel-group randomized controlled trial (1:1 ratio) among Moroccan pilgrims registered for the 2025 pilgrimage, comparing a pre-departure therapeutic patient education program with no education, and reported it according to the Consolidated Standards of Reporting Trials (CONSORT) 2025 statement [14]. The study took place in the vaccination centers of Salé and Rabat dedicated to departing pilgrims, with recruitment from 15 to 18 April 2025, a short window dictated by pilgrims' travel schedule that required a large team of physicians for participant recruitment, eligibility screening, and intervention delivery and training. Follow-up comprised four measurement times defined in relation to each participant's travel milestones: T0, at enrollment; T1, seven days before departure; T2, 48 hours after the end of the peak activity period; and T3, seven days after return. The peak activity period, the phase of maximal walking load, ran on fixed dates for all participants (4-9 June 2025), so T2 fell on the same calendar date (11 June 2025) in both groups. Median delays since enrollment were 25 days for T1, 56 for T2, and 71 for T3.

Participants

Eligible participants were adults aged 50-75 years registered for the 2025 pilgrimage. We excluded participants with active inflammatory joint disease, a lower limb joint prosthesis, a contraindication to prolonged walking, or a cognitive or psychiatric disorder preventing understanding of or adherence to the protocol. Participants were recruited during the pre-vaccination consultation, with standardized oral and written information and eligibility checked before inclusion.

Randomization and blinding

An independent statistician not involved in enrollment generated the allocation sequence with jamovi (version 2.7.29.0), using simple 1:1 randomization without stratification. Allocation was concealed by keeping the sequence with the independent statistician, who communicated each assignment by telephone or message on request, after eligibility had been confirmed and written consent obtained. Blinding of participants and providers was not feasible for a therapeutic patient education program. Outcome assessment at T1, T2, and T3 was carried out blind by two independent assessors uninvolved in recruitment or the intervention, with no access to the randomization list. Statistical analysis used groups coded "A" and "B", with unblinding after the analysis was complete.

Intervention

This therapeutic patient education program comprised a face-to-face group education session, an unsupervised home-based adapted physical activity program, and WhatsApp reminders reinforcing the key messages. The intervention group attended a face-to-face group education session of around one hour, combined with an unsupervised home-based adapted physical activity program. Sessions were held in small groups of four to five participants (10 at most), run by two trained rheumatology residents, and covered four areas: preparation before departure (choice and fitting of footwear, clothing, packing of the backpack); prevention and management of lower limb and spinal pain by physical means (cryotherapy, orthoses, elastic compression, lumbar support belts); strategies during the stay (ergonomics, protective and active postures, alternating effort and rest, cane use, hydration, fatigue management); and fall prevention. The home-based program comprised progressive walking and daily 15-20-minute exercises covering warm-up, stretching, strengthening, and balance.

Content was illustrated with photographs, and exercises were presented as video capsules given to participants with all audiovisual materials. Two WhatsApp reminders, restating the key messages of the four session areas and resending these materials, were sent one week before departure and two days after arrival. All intervention participants received identical content, both residents delivering the sessions from the same standardized session outline and with the same photographs and video capsules; the control group received no specific intervention. Adherence and fidelity were not formally assessed, and no monitoring tool or threshold was defined a priori.

Outcomes

The primary outcome was the VAS score (0-10) for mean overall lower limb pain felt during the day of assessment at T2, with no anatomical location specified. Secondary outcomes, measured at T2 unless stated otherwise, were the same scale for low back pain; the location of maximal lower limb pain (hip, knee, ankle, or foot); quality of life measured with the EuroQol 5-dimension 5-level questionnaire (EQ-5D-5L) [15]; increased analgesic use, defined as any self-reported rise in the frequency or dose of paracetamol and/or NSAIDs for lower limb or low back pain compared with T0; at least one consultation for lower limb pain and for low back pain; limping; lower limb muscle cramps; and at least one fall. All secondary outcomes were binary except the pain scores and the location of maximal pain. Adverse events related to the home-based physical activity program were not systematically collected.

Data collection

T0 data were collected face-to-face after the clinical examination. T1, T2, and T3 data were collected remotely, by telephone or WhatsApp, by the two blinded assessors, both rheumatology residents.

At enrollment, we recorded planned departure and return dates, age, gender, self-reported history and comorbidities, and habitual analgesic use for joint pain with drug class. Weight and height were measured with calibrated equipment to calculate body mass index (BMI). The musculoskeletal examination, carried out by the including resident, looked for limping, lumbar spine stiffness (non-zero finger-to-floor distance), knee effusion, knee flexion contracture, hip flexion contracture, and restricted ankle dorsiflexion. Physical activity level was collected with a single four-option question: never, occasional, three times per week, or daily.

Pain was rated from 0 (no pain) to 10 (worst pain), separately for the lower limbs and the lumbar spine, at all four times. At T0, the scale was self-administered under supervision; at T1, T2, and T3, the participant reported the numerical value by telephone or WhatsApp. All participants received a structured one-hour introduction to the scale at recruitment, with explanations, examples, a check of understanding by rephrasing, and an explanatory video in Moroccan dialect sent by WhatsApp.

Quality of life was assessed at T0 and T2 with the EQ-5D-5L Morocco (Arabic), Paper Interviewer Administration, version 1.3 (EuroQol Research Foundation, Rotterdam, the Netherlands) [16], administered orally with a standardized procedure. Its descriptive system covers five dimensions with five ordered levels each, from 1 (no problem) to 5 (extreme problems). The utility index was derived from the Moroccan value set [17].

The post-travel questionnaire recorded limping and lower limb muscle cramps at T2; healthcare use, defined as at least one consultation for lower limb pain or for low back pain at a health center or in the emergency department; increased analgesic use as defined above; and at least one fall of any severity. Numbers and circumstances of consultations and falls were not recorded. Exposure conditions were recorded as ordered categories: mean daily walking time, daily time spent at the main gathering site, usual sitting posture during prolonged seated periods, and footwear mainly worn. The accommodation-to-site distance was estimated with the Google Maps walking route.

A standardized script was used to administer the pain scale and the post-travel questionnaire, with a maximum five-day window per time point: a call on the first day; if unanswered, a WhatsApp audio message accepting an audio reply; and then daily attempts until the measure was obtained, limiting missing data and recall bias. Answers were entered into a spreadsheet database with consistency checks and preset coding, anonymized with a unique identifier, without name or group, and accessible only to the two assessors.

Sample size

Sample size was calculated with jamovi (version 2.7.29.0) for a two-sided independent-samples t-test on the primary outcome. In the absence of pilot data in this population, we targeted a minimal clinically important between-group difference of 2 points on the pain scale (α=5%) [18], corresponding to a small-to-medium standardized effect size (Cohen's d≈0.4) [19]. With 80% power, 99 participants per group were required; this number was inflated by approximately 10% to anticipate loss to follow-up and to preserve adequate power for secondary outcomes, yielding a target of 109 participants per group (218 total), consistent with final enrollment.

Human rights and ethical considerations

All procedures involving human participants accorded with the ethical standards of the institutional research committee and with the 1964 Declaration of Helsinki and its later amendments [20]. The protocol was approved by the Biomedical Research Ethics Committee of Mohammed V University, Rabat, Morocco (approval number: CERB 69-25; approval date: 9 April 2025). The trial was registered in the Pan African Clinical Trial Registry (ID number: PACTR202506664204956). All participants gave written informed consent after oral and written information.

Statistical analysis

Baseline characteristics were described by group as mean (standard deviation), median (interquartile range) when not normally distributed, or number (%). We analyzed the primary outcome with a linear mixed model for repeated measures, including group, time (visit), and the group by time interaction as fixed effects, and a random intercept per participant. Simple group effects (education minus control) were estimated at each visit (T0-T3) with 95% confidence intervals (CIs), degrees of freedom being approximated with the Satterthwaite method. An adjusted analysis then used prespecified covariates: age, gender, BMI, physical activity level, daily walking time, accommodation-to-site distance, and footwear type. Low back pain was analyzed with the same model, unadjusted and adjusted for these covariates plus sitting posture and daily time at the site. The EQ-5D-5L utility index used the same model structure at T0 and T2, unadjusted and adjusted for age, gender, and BMI, with Wald CIs, covering 432 observations from 216 participants. Baseline characteristics were compared between groups with the Student t-test (normally distributed continuous variables), the Mann-Whitney U test (other continuous variables), and the Pearson chi-squared test or the Fisher exact test (categorical variables). Binary secondary outcomes were compared with the Pearson chi-squared test or the Fisher exact test. Analyses followed intention to treat, including every randomized participant with at least one available measure. Missing outcome data were not imputed: the linear mixed models used all available observations, which yields valid estimates under the assumption that data are missing at random. The two control participants without a T2 measurement therefore contributed their earlier observations. Binary secondary outcomes were analyzed in the participants with available T2 data (n=216). Model assumptions were checked by visual inspection of residual Q-Q plots and residuals-versus-predicted plots for each model; residuals were approximately normally distributed with no evidence of heteroscedasticity, apart from a departure in the tails of the low back pain model consistent with the floor effect of that outcome. Secondary outcomes were neither ranked nor adjusted for multiple comparisons and were interpreted as exploratory. Significance was set at p<0.05, two-sided. Analyses used jamovi version 2.7.29.0 (the jamovi project, Sydney, Australia) [21].

## Results

Participant flow

In total, 218 pilgrims were randomized, 109 to the education group and 109 to the control group (Figure 1). Two control participants had no lower limb pain measure at T2; they contributed their earlier measures to the primary analysis.

**Figure 1:**
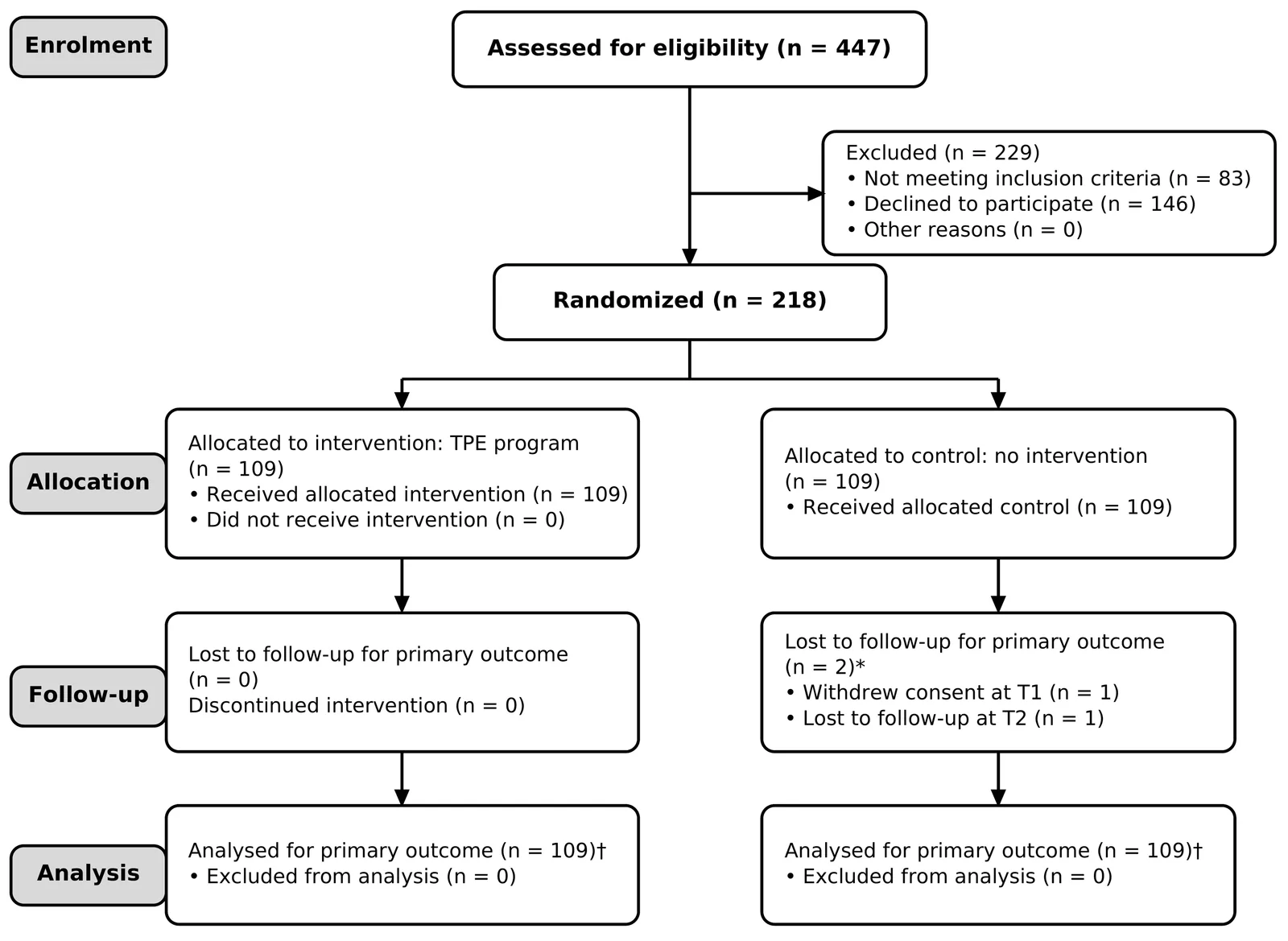
Participant flow diagram *In the control group, two participants had no lower limb pain (VAS) measurement at T2, the primary outcome time point. †Primary outcome analyzed with a linear mixed model on an intention-to-treat basis, including all randomized participants with at least one available measurement; the two control participants without a T2 measurement contributed their earlier observations (n=107 with a T2 measurement). TPE: therapeutic patient education; VAS: visual analog scale; T1: seven days before departure; T2: 48 hours after the end of the peak activity period

Baseline and clinical characteristics

Groups were comparable at baseline (Table 1). The mean age was 62.6 (6.1) years, and 147 (67.4%) participants were women. The mean BMI was 28.5 (4.5) kg/m²: 87 (39.9%) were overweight, and 83 (38.1%) were obese. Lower limb pain was present in 112 (51.6%) participants and low back pain in 66 (30.4%). At enrollment, the median lower limb pain was 3.0 (interquartile range: 0.0-5.0), and the median low back pain was 0.0 (0.0-3.0). The main comorbidities were hypertension (59, 27.1%) and diabetes (47, 21.6%). For joint pain, 123 (56.4%) took paracetamol, and 78 (35.8%) took an NSAID. Physical activity level was low, with 32 (14.7%) never active and 99 (45.4%) occasionally active. On examination, lumbar spine stiffness was found in 167 (76.6%), whereas other abnormalities were infrequent: knee effusion 13 (6%), limping 7 (3.2%), and knee flexion contracture 7 (3.2%).

**Table 1:** Baseline and clinical characteristics of the study population Symbols preceding a characteristic indicate how that variable is expressed; letters following a value in the "Test statistic value" column indicate which test produced it. *Mean (standard deviation); intervention minus control. †Number (percentage). ‡Median (Q1-Q3); quartiles were calculated by linear interpolation; for both baseline pain scores, n=109 in the intervention group and n=108 in the control group; pain was rated on a visual analog scale from 0 to 10. §Lumbar spine stiffness assessed by a non-zero finger-to-floor distance. Percentages use available observations; one control participant has missing baseline lower limb and low back pain data. ^a^Two-sided independent-samples Student t-test with pooled variance (intervention minus control; 216 degrees of freedom). ^b^Pearson chi-squared test without continuity correction (1 degree of freedom, except physical activity level, 3 degrees of freedom). ^c^Two-sided Mann-Whitney U test using an asymptotic p-value corrected for ties and continuity; U is reported for the intervention group. ^d^Fisher exact test, used because expected cell counts were <5; no test statistic is associated with this test. ^e^Not estimable: both groups had zero events. Body mass index categories: underweight <18.5 kg/m²; normal weight 18.5-24.9 kg/m²; overweight 25.0-29.9 kg/m²; and obesity ≥30 kg/m². Intervention: therapeutic patient education. NSAID: nonsteroidal anti-inflammatory drug; T0: enrollment

Characteristic	Intervention (n=109)	Control (n=109)	Total (n=218)	Test statistic value	P-value
Baseline characteristics					
Age, years*	62.5 (6.0)	62.8 (6.2)	62.6 (6.1)	-0.3016^a^	0.763
Female gender†	74 (67.9%)	73 (67%)	147 (67.4%)	0.02^b^	0.89
Body mass index, kg/m²*	28.5 (4.7)	28.5 (4.3)	28.5 (4.5)	0.0939^a^	0.925
Underweight†	0 (0%)	1 (0.9%)	1 (0.5%)		
Normal weight†	20 (18.3%)	27 (24.8%)	47 (21.6%)		
Overweight†	51 (46.8%)	36 (33%)	87 (39.9%)		
Obesity†	38 (34.9%)	45 (41.3%)	83 (38.1%)		
Lower limb pain†	56 (51.4%)	56 (51.9%)	112 (51.6%)	0.00^b^	0.94
Low back pain†	35 (32.1%)	31 (28.7%)	66 (30.4%)	0.30^b^	0.59
Lower limb pain score at T0‡	3.00 (0.00-5.00)	3.00 (0.00-5.00)	3.00 (0.00-5.00)	5955^c^	0.875
Low back pain score at T0‡	0.00 (0.00-3.00)	0.00 (0.00-3.25)	0.00 (0.00-3.00)	5970^c^	0.824
Hypertension†	27 (24.8%)	32 (29.4%)	59 (27.1%)	0.58^b^	0.45
Diabetes†	24 (22%)	23 (21.1%)	47 (21.6%)	0.03^b^	0.87
NSAID use for joint pain†	40 (36.7%)	38 (34.9%)	78 (35.8%)	0.08^b^	0.78
Paracetamol use for joint pain†	66 (60.6%)	57 (52.3%)	123 (56.4%)	1.51^b^	0.22
Physical activity level†					
Never	14 (12.8%)	18 (16.5%)	32 (14.7%)	1.40^b^	0.71
Occasional	53 (48.6%)	46 (42.2%)	99 (45.4%)		
3 times per week	23 (21.1%)	22 (20.2%)	45 (20.6%)		
Daily	19 (17.4%)	23 (21.1%)	42 (19.3%)		
Clinical characteristics					
Limping†	4 (3.7%)	3 (2.8%)	7 (3.2%)	_^d^	1.00
Lumbar spine stiffness†§	85 (78%)	82 (75.2%)	167 (76.6%)	0.23^b^	0.63
Knee effusion†	8 (7.3%)	5 (4.6%)	13 (6%)	0.74^b^	0.39
Knee flexion contracture†	4 (3.7%)	3 (2.8%)	7 (3.2%)	_^d^	1.00
Hip flexion contracture†	0 (0%)	0 (0%)	0 (0%)	NA^e^	NA^e^
Restricted ankle dorsiflexion†	0 (0%)	0 (0%)	0 (0%)	NA^e^	NA^e^

Primary outcome

Lower limb pain did not differ between groups at enrollment (T0: +0.08; 95% CI: −0.58 to 0.73; p=0.815). A difference favoring the education group appeared at T1 (−0.69; 95% CI: −1.34 to −0.03; p=0.039), widened at T2 (−1.75; 95% CI: −2.40 to −1.09; p<0.001), and was maintained at T3 (−1.65; 95% CI: −2.31 to −1.00; p<0.001) (Table 2, Figure 2a). The point estimate at T2 did not reach the prespecified 2-point threshold of clinical importance. Adjusted results were consistent (T1: −0.66; 95% CI: −1.29 to −0.03; p=0.040; T2: −1.72; 95% CI: −2.35 to −1.09; p<0.001; T3: −1.62; 95% CI: −2.25 to −1.00; p<0.001) (Figure 2b).

**Table 2:** Effect of the intervention on pain and quality of life (linear mixed models) *Between-group difference (intervention minus control) in visual analog scale points (0-10), estimated with a linear mixed model including group, time, and the group by time interaction; a negative value indicates lower pain in the intervention group. The lower limb pain model was adjusted for age, gender, body mass index, physical activity level, daily walking time, accommodation-to-site distance, and footwear type; the low back pain model additionally included sitting posture and daily time at the main gathering site. †Between-group difference in the EQ-5D-5L index (range: 0-1); a positive value indicates better health-related quality of life in the intervention group. The EQ-5D-5L was assessed at T0 and T2 only. P-values for the available adjusted lower limb contrasts are from two-sided t-tests of simple group effects in the linear mixed model, using Satterthwaite degrees of freedom. Bold p-values indicate p<0.05. CI: confidence interval; EQ-5D-5L: EuroQol 5-dimension 5-level questionnaire; T0 :enrollment; T1: seven days before departure; T2: 48 hours after the end of the peak activity period; T3: seven days after return

Outcome and time point	Unadjusted difference (95% CI)	P-value	Adjusted difference (95% CI)	P-value
Lower limb pain score*				
T0	+0.08 (−0.58 to 0.73)	0.815	+0.11 (−0.52 to 0.74)	0.728
T1	−0.69 (−1.34 to −0.03)	0.039	−0.66 (−1.29 to −0.03)	0.040
T2	−1.75 (−2.40 to −1.09)	<0.001	−1.72 (−2.35 to −1.09)	<0.001
T3	−1.65 (−2.31 to −1.00)	<0.001	−1.62 (−2.25 to −1.00)	<0.001
Low back pain score*				
T0	−0.005 (−0.59 to 0.58)	0.987	+0.12 (−0.48 to 0.72)	0.687
T1	+0.09 (−0.50 to 0.68)	0.760	+0.22 (−0.38 to 0.82)	0.466
T2	−0.46 (−1.05 to 0.13)	0.123	−0.33 (−0.93 to 0.27)	0.279
T3	−0.46 (−1.05 to 0.13)	0.123	−0.33 (−0.93 to 0.27)	0.278
EQ-5D-5L index†				
T0	+0.019 (−0.031 to 0.069)	0.449	+0.020 (−0.030 to 0.069)	0.436
T2	+0.059 (0.009 to 0.109)	0.021	+0.059 (0.010 to 0.108)	0.019

**Figure 2:**
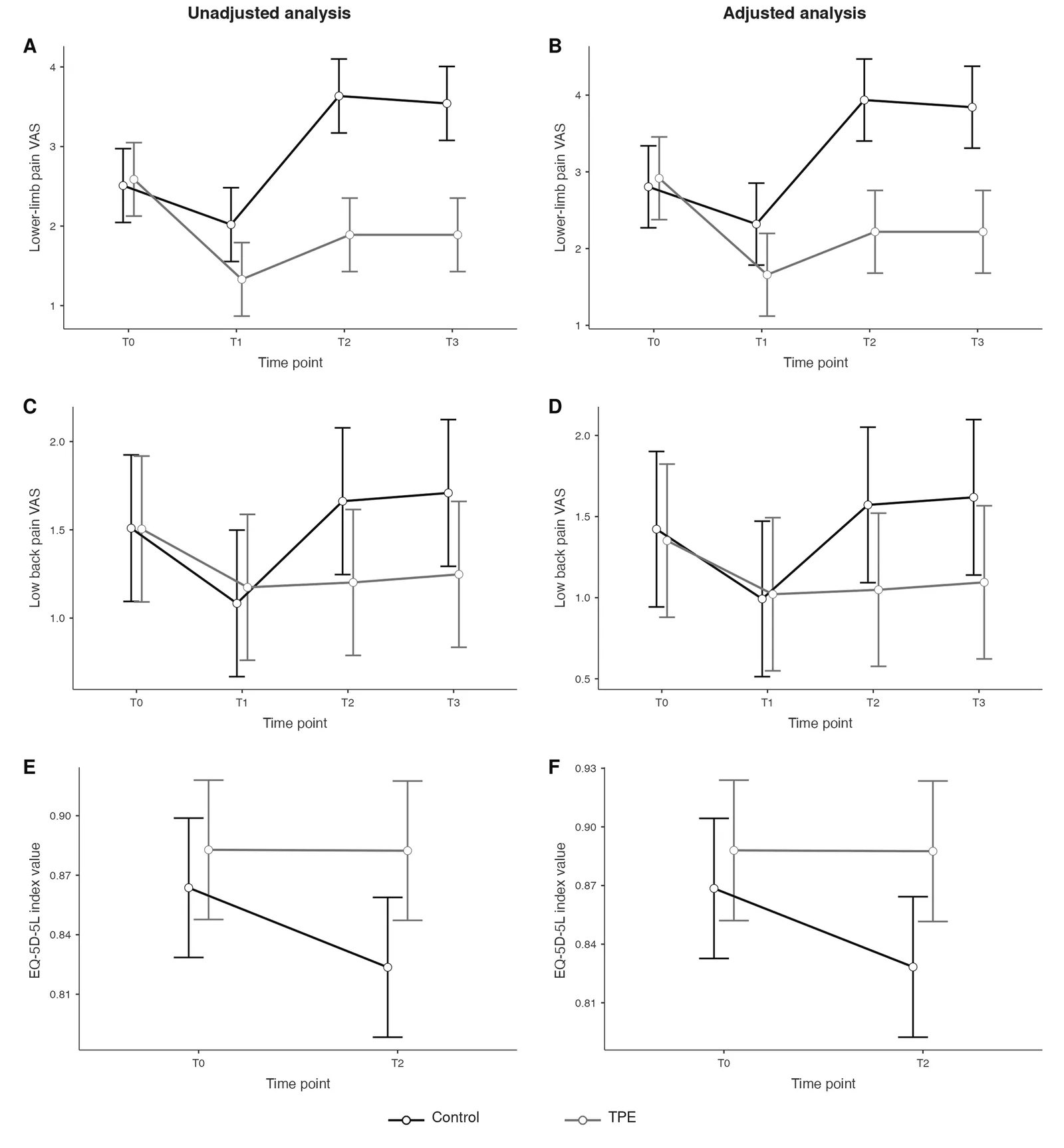
Effect of therapeutic patient education versus control on clinical outcomes over time (A) Lower limb pain, unadjusted analysis. (B) Lower limb pain, adjusted analysis. (C) Low back pain, unadjusted analysis. (D) Low back pain, adjusted analysis. (E) Health-related quality of life (EQ-5D-5L index), unadjusted analysis. (F) Health-related quality of life (EQ-5D-5L index), adjusted analysis. Pain was rated on a visual analog scale from 0 to 10. EQ-5D-5L: EuroQol 5-dimension 5-level questionnaire; T0: enrollment; T1: seven days before departure; T2: 48 hours after the end of the peak activity period; T3: seven days after return

Location of maximal pain, limping, and cramps

At T2, maximal pain was less often located in the foot in the education group (11 (10.1%) versus 24 (22.4%); p=0.01) (Table 3). Differences for the knee (42 (38.5%) versus 53 (49.5%); p=0.10), ankle (8 (7.3%) versus 9 (8.4%); p=0.77), and hip (3 (2.8%) versus 7 (6.5%); p=0.19) were not significant. Limping was less frequent in the education group (24 (22%) versus 42 (39.3%); p=0.01), whereas muscle cramps did not differ (19 (17.4%) versus 29 (27.1%); p=0.09).

**Table 3:** Location of maximal lower limb pain and secondary binary outcomes during the stay Values are number (percentage). Percentages were calculated using analyzed participants as the denominator. The letter following each test statistic value indicates the test used, as in Table 1. ^b^Two-sided Pearson chi-squared test without continuity correction (1 degree of freedom); no other test was used in this table. Bold p-values indicate p<0.05. Intervention: therapeutic patient education. T2: 48 hours after the end of the peak activity period

Outcome	Total (n=216)	Intervention (n=109)	Control (n=107)	Test statistic value	P-value
Location of maximal lower limb pain at T2					
Knee	95 (44%)	42 (38.5%)	53 (49.5%)	2.65^b^	0.10
Foot	35 (16.2%)	11 (10.1%)	24 (22.4%)	6.05^b^	0.01
Ankle	17 (7.9%)	8 (7.3%)	9 (8.4%)	0.09^b^	0.77
Hip	10 (4.6%)	3 (2.8%)	7 (6.5%)	1.76^b^	0.19
Secondary binary outcomes during the stay					
Limping (T2)	66 (30.6%)	24 (22%)	42 (39.3%)	7.56^b^	0.01
Lower limb muscle cramps (T2)	48 (22.2%)	19 (17.4%)	29 (27.1%)	2.92^b^	0.09
Increased analgesic use	81 (37.5%)	33 (30.3%)	48 (44.9%)	4.90^b^	0.027
Consultation for lower limb pain	48 (22.2%)	21 (19.3%)	27 (25.2%)	1.11^b^	0.29
Consultation for low back pain	4 (1.9%)	1 (0.9%)	3 (2.8%)	1.06^b^	0.30
Fall	10 (4.6%)	5 (4.6%)	5 (4.7%)	0.00^b^	0.98

Low back pain

Low back pain scores were lower in the education group at T2 and T3 without reaching significance (−0.46; 95% CI: −1.05 to 0.13; p=0.123 at both times), and the adjusted result was consistent (−0.33; 95% CI: −0.93 to 0.27; p=0.279) (Table 2, Figure 2c-2d).

Quality of life

The EQ-5D-5L index did not differ between groups at enrollment (+0.019; 95% CI: −0.031 to 0.069; p=0.449). At T2, quality of life was better preserved in the education group (+0.059; 95% CI: 0.009 to 0.109; p=0.021 unadjusted; +0.059; 95% CI: 0.010 to 0.108; p=0.019 after adjustment for age, gender, and BMI) (Table 2, Figure 2e-2f). Dimension-level transitions across the five EQ-5D-5L domains are shown in Figure 3.

**Figure 3:**
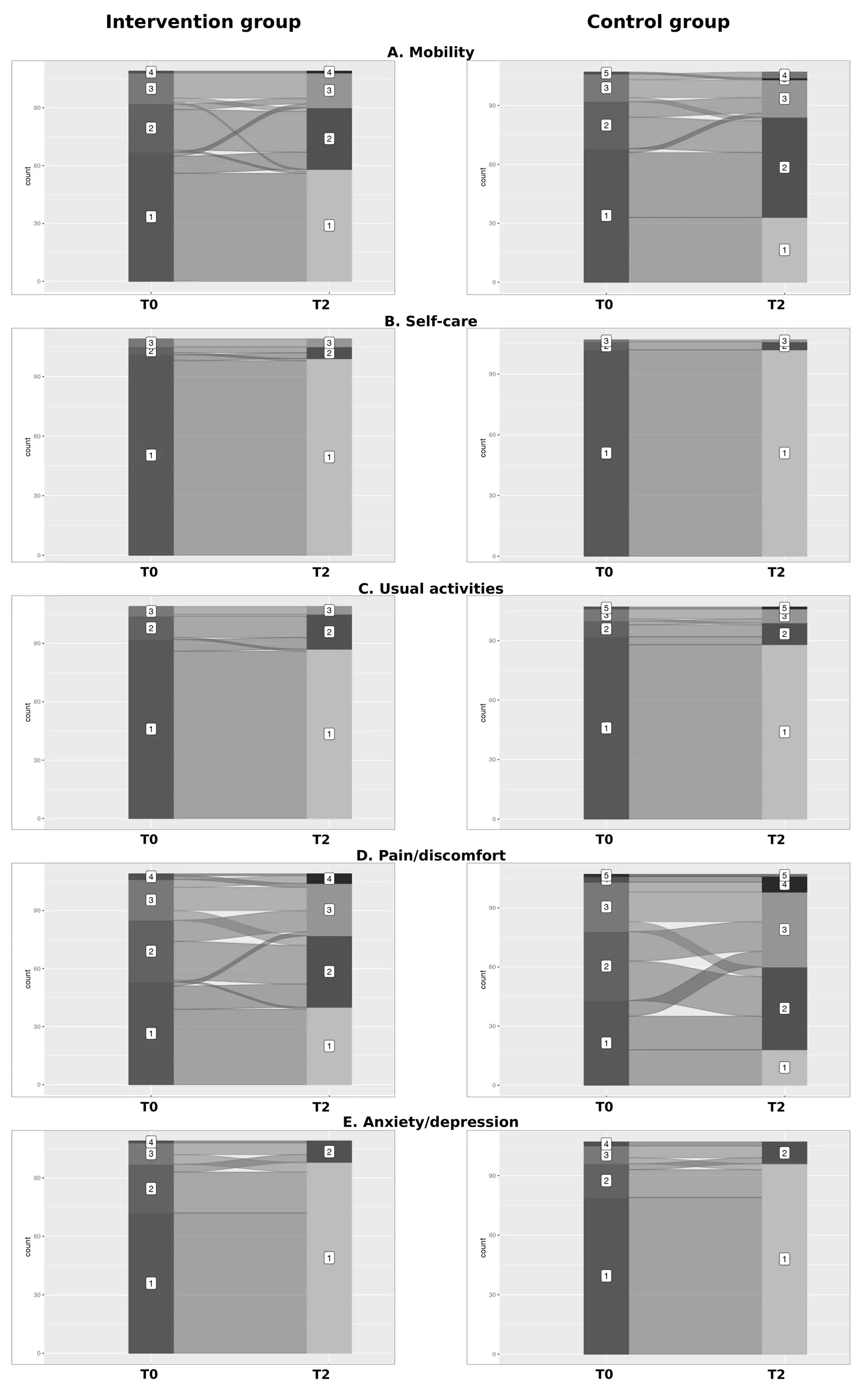
Individual transitions in EQ-5D-5L dimension severity levels from T0 to T2 in the intervention and control groups EQ-5D-5L: EuroQol 5-dimension 5-level questionnaire; T0: enrollment; T2: 48 hours after the end of the peak activity period

Analgesic use, healthcare use, and falls

Increased analgesic use was less frequent in the education group (33 (30.3%) versus 48 (44.9%); p=0.027) (Table 3). Consultations did not differ, either for lower limb pain (21 (19.3%) versus 27 (25.2%); p=0.29) or for low back pain (1 (0.9%) versus 3 (2.8%); p=0.30). Falls did not differ (5 (4.6%) versus 5 (4.7%); p=0.98).

## Discussion

In this randomized controlled trial in 218 adults aged 50-75 years exposed to several days of intense unaccustomed walking, a structured education program delivered before departure reduced lower limb pain intensity. At T2, the between-group difference was −1.75 points out of 10 (95% CI: −2.40 to −1.09; p<0.001), and the adjusted analysis was consistent (−1.72; 95% CI: −2.35 to −1.09). The point estimate did not reach the 2-point threshold set a priori, but the CI contains values compatible with a clinically important benefit, so the trial can neither confirm nor exclude one. The effect appeared at T1, before exposure, widened during it, and persisted seven days after return. Exploratory secondary outcomes varied consistently, with less limping, less increased analgesic use, and less frequent foot pain, whereas no difference was found for low back pain, healthcare use, or falls.

The intervention combined protective behavioral strategies with an unsupervised home-based adapted physical activity program taught during the single session and illustrated by video capsules, so it was neither purely informative education nor education combined with supervised exercise. In the systematic review by Goff et al. [22], education alone modestly reduced short-term pain (standardized mean difference: −0.35; low certainty) and was more effective combined with exercise. The meta-analysis by Cormier et al. [13], covering 69 randomized trials in subacute and chronic musculoskeletal conditions, was convergent: adding education to other interventions probably reduced pain, disability, and catastrophizing in the short term (moderate certainty), whereas its stand-alone efficacy remained uncertain. These estimates do not transfer directly to our intervention, whose exercise component was taught within the session and then performed without supervision.

Among available trials, that of Jinnouchi et al. [23] is closest to our program: a single 100-minute session combining self-exercise learning and concise pain education improved function and self-efficacy at 12 weeks, without effect on pain intensity or the EQ-5D. Two elements may explain why our results differ. The first is the comparator: our control group received no intervention, whereas trials using an active comparator matched for duration, format, and setting, such as that of Darnall et al. [24] (−1.02 points; 95% CI: −1.60 to −0.43), estimate a narrower effect specific to educational content. The second is the target: our content aimed at preventing a predictable and dated mechanical load in participants not selected for chronic pain, rather than at managing established pain.

These results should also be placed within the mass-gathering literature, where the need is documented but the preventive response is rarely evaluated. Alshahrani et al. [25] found musculoskeletal conditions to be the second diagnostic category of healthcare contacts during the 2025 pilgrimage (24.5% in primary care), with complaints peaking toward the end of the stay. The scoping review by Zaini et al. [26] identified only 17 studies of health education in mass gatherings, including one randomized trial, none assessing morbidity or quality of life during the event itself. Our trial adds a randomized estimate on a clinical outcome measured during the event.

The difference in lower limb pain at T1 (−0.69 points), before exposure, may reflect an early effect of physical preparation, an expectation bias, or both. Three elements limit this concern. First, the gap widened markedly during exposure (−1.75 at T2), which a time-stable expectation bias would explain poorly. Second, the intervention group reported less pain while also reporting less increased analgesic use (33 (30.3%) versus 48 (44.9%)), whereas simple reporting compliance would rather have left consumption comparable. Third, the foot, the site directly targeted by footwear advice, was the only location reaching significance (11 (10.1%) versus 24 (22.4%); p=0.01), a content-result consistency that remains exploratory. Plausible but unmeasured mechanisms include graded exposure before departure and footwear choice, load management, and protective postures during the stay; persistence of the effect after return is compatible with maintained self-management.

The lower frequency of limping at T2 (24 (22%) versus 42 (39.3%); p=0.01) points in the same direction. Limping is a functional outcome, less directly dependent on self-rated pain intensity, and its concordance with less frequent foot pain strengthens the internal coherence of the signal, without these unranked comparisons being confirmatory. The lower frequency of increased analgesic use is an exploratory result of public health relevance, since NSAIDs are among the classes most often prescribed in this setting [9] and are used here amid heat, prolonged effort, and dehydration, a combination exposing to misuse and acute kidney injury [10,11]. No clinical benefit from lower anti-inflammatory exposure was measured directly.

Less pain and less analgesic use were not accompanied by a difference in healthcare use (21 versus 27 participants). Samarkandi et al. [27] reported that 59.9% of participants surveyed after the 2024 pilgrimage had not sought medical help despite severe symptoms. Consultation numbers reflect access to care and help-seeking behavior as much as symptom burden, so this result is uninterpretable rather than evidence of no effect.

For low back pain, the 95% CI of the difference at T2 (−1.05 to +0.13) excludes a benefit of at least 2 points, an informative negative result rather than an inconclusive one. The trial was not designed to treat active low back pain: only 30.4% of participants had low back pain at enrollment, and the median score was zero, creating a structural floor effect. The favorable results obtained in selected patients by Kim et al. [28] and Pocovi et al. [29] do not contradict ours, their populations and outcomes differing.

At the dimension level, the EQ-5D-5L data add detail to the utility difference observed at T2. In both groups, participants shifted toward more severe levels for mobility and for pain or discomfort, but the shift was greater in controls (level 1 proportion: mobility, 61.5% to 53.2% in the education group versus 63.6% to 30.8% in controls; pain or discomfort, 48.6% to 36.7% versus 40.2% to 16.8%). Usual activities deteriorated slightly, and self-care remained stable in both groups, whereas anxiety or depression improved markedly in both (level 1 proportion rising from 66.1% to 89.9% and from 73.8% to 89.7%, respectively). The divergence was thus concentrated in the two dimensions most tied to musculoskeletal load, whereas dimensions less dependent on lower limb function changed little. The parallel improvement in anxiety or depression cannot be attributed to the program and more plausibly reflects a non-specific effect of completing a long-anticipated, demanding journey. These changes were neither prespecified nor formally tested between groups.

In practical terms, the program is short, requires no specialized equipment, and fits into the existing pre-departure consultation; since the physical activity is unsupervised, it needs neither a rehabilitation facility nor supervised sessions. This matters where around 34,000 travellers [5], many with musculoskeletal symptoms and metabolic comorbidity, pass each year through an already organized medical contact point. Delivery in the country of origin, in Moroccan dialect and with visual materials, matches the recommendations for mass gatherings [26] and suits a population with heterogeneous literacy. More broadly, preventive education timed to a foreseeable rise in ambulatory load could apply to other planned exposures, such as organized group travel or long-distance walking events. The format we evaluated, run by resident physicians in small groups, cannot however be considered immediately scalable, and no cost data were collected.

Study limitations

First, the absence of an active control group matched for contact time is the main threat to internal validity, since contact time, group dynamics, and reminders cannot be separated from educational content, nor can the respective contributions of the education session, the home-based physical activity program, and the WhatsApp reminders be separated from one another; the trial therefore estimates the overall effect of the program as delivered. Traeger et al. [30] and Darnall et al. [24] illustrate this distinction, a matched comparator substantially changing the estimate of the educational effect. Second, participant blinding was not feasible, and the primary outcome relied on pain intensity reported by telephone. Third, secondary comparisons were neither ranked nor adjusted for multiplicity and remain exploratory, including the EQ-5D-5L estimate. Fourth, no systematic adverse-event surveillance was set up, so the tolerability of the home-based adapted physical activity program cannot be established here; Pocovi et al. [29] reported more lower limb adverse events in their intervention group (100 versus 54). Fifth, 146 of the 447 adults assessed for eligibility declined to participate, and exposure conditions were recorded as self-reported ordered categories, so selection effects and residual confounding cannot be excluded. Sixth, the sample was sized to detect a 2-point difference, which limits the precision available around smaller effects. Finally, adherence and fidelity were not formally assessed, and recruitment in only two centers of a single national contingent, in a population aged 50-75 years and mainly women, limits extrapolation to men, to younger participants, and to other settings.

Study strengths

The trial rests on an intention-to-treat analysis, an independently generated allocation sequence, and a linear mixed model over four measurement times, with blinded assessment and analysis. Follow-up quality is a particular asset: the primary outcome was obtained for 216 of 218 participants (99.1%) during a mass gathering, through a reproducible procedure combining a five-day window, daily reminders, and WhatsApp audio messaging. Finally, pain intensity, limping, analgesic use, and health utility varied in a consistent direction, strengthening the coherence of the signal without turning each secondary comparison into confirmatory evidence.

## Conclusions

In conclusion, among adults aged 50-75 years exposed to several days of intense unaccustomed walking, a therapeutic patient education program delivered before departure produced a statistically significant reduction in lower limb pain intensity, with a point estimate below the 2-point threshold set a priori. The trial can neither confirm nor exclude a clinically important benefit.

Short and resource-light, this intervention may be a realistic preventive option for adults facing a predictable episode of high ambulatory load in comparable populations, but its specific educational effect, adherence, and safety remain to be established. Confirmation requires multicenter trials with an active comparator matched for contact time, measurement of adherence, fidelity, and adverse events and longer follow-up.
